# The relationship between HYDIN and fallopian tubal cilia loss in patients with epithelial ovarian cancer

**DOI:** 10.3389/fonc.2024.1495753

**Published:** 2025-01-09

**Authors:** Yuanli Guo, Xinxin He, Junfeng Liu, Yanming Tan, Chao Zhang, Shan Chen, Sheng Zhang

**Affiliations:** ^1^ Department of Pathology, The First Affiliated Hospital of Fujian Medical University, Fuzhou, China; ^2^ Department of Obstetrics and Gynecology, The First Affiliated Hospital of Guangdong Pharmaceutical University, Guangzhou, China; ^3^ Department of Pathology, Sun Yat-sen Memorial Hospital of Sun Yat-sen University, Guangzhou, China; ^4^ Department of Pathology, The First Affiliated Hospital of Sun Yat-sen University, Guangzhou, China; ^5^ GenePlus-Shenzhen, Shenzhen, China; ^6^ Department of Gynecology, The Six Affiliated Hospital, Sun Yat-sen University, Guangzhou, China; ^7^ Biomedical Innovation Center, The Sixth Affiliated Hospital, Sun Yat-sen University, Guangzhou, China

**Keywords:** fallopian tubes, cilia, epithelial ovarian cancer, HYDIN, marker

## Abstract

**Introduction:**

Primary cilia play an important role in the development of cancer by regulating signaling pathways. Several studies have demonstrated that women with *BRCA* mutations have, on average, 50% fewer ciliated cells compared with general women. However, the role of tubal cilia loss in the development of epithelial ovarian cancer (EOC) remains unclear. Few specific studies have been found in linking *HYDIN*, a ciliary defect associated gene that encodes *HYDIN* axonemal central pair apparatus protein, which is involved in the transduction of Hedgehog (Hh) signal and is considered a cancer associated antigen, to ovarian cancer. Therefore, our study aimed to investigate the correlation between *HYDIN* gene mutations and tubal cilia loss in EOC.

**Methods:**

A whole exome sequencing (WES), immunohistochemistry (IHC), western blot, and reverse transcription quantitative (RT q) PCR were performed in 80 patients with EOC and 50 cases of non ovarian cancer to detect the mutations and expression of tubal ciliary marker, ciliary morphology, and abnormal rate.

**Results:**

We found that the incidence of tubal cilia loss was higher in EOC group with decreased expression of *HYDIN* compared with the control group (P<0.05).

**Discussion:**

This study suggests that tubal ciliary loss is evident in epithelial fallopian tube carcinoma, and ciliary cells may be involved in the occurrence and development of EOC, and cilia-related gene *HYDIN* is expected to be a tumor marker for epithelial ovarian cancer.

## Introduction

Ovarian cancer is the seventh most common cancer in women worldwide due to its atypical early clinical signs and symptoms. According to statistics, there are about 1.34 million new cases of gynecological cancer and about 550,000 deaths in 2020. Although the incidence of ovarian cancer is lower than that of cervical cancer and endometrial cancer, it has the worst prognosis and the highest mortality (1). Due to the lack of clear screening tools, about 75% of patients were diagnosed with ovarian cancer at an advanced stage. The most common type of ovarian cancer is epithelial ovarian cancer (EOC), accounting for 90% to 95% of all ovarian cancers (2, 3). EOC is highly heterogeneous, with different subtypes showing diversity of biological behavior and unique morphological and molecular heritages EOC is characterized by its strong ability to invade surrounding tissues and rapid growth (4). Studies have shown that most EOC origin sites are located in the transition area between the fallopian tube and the peritoneal epithelium in the fimbria part of the fallopian tube (5). Therefore, it is important to investigate the multiple signaling pathways and underlying mechanisms associated with the occurrence of ovarian cancer to identify novel therapeutic targets for EOC treatment, improve the clinical efficacy of ovarian cancer treatment, and reduce the recurrence rate of cancer. Studies have shown that there are cilia on the fallopian tube. Cilia are microtubule structures extending from the centrosome, which can be divided into motile cilia and non-motile cilia (primary cilia) (6). Previous studies have shown that primary cilia exist in a variety of tumors, and a variety of ligands, such as Hedgehog and Wnt, and Notch receptor are involved in signal transduction on the ciliary membrane. These primary cilia-mediated signaling pathways are critical for cell survival, proliferation, differentiation, and death, and are involved in paracellular signaling between cancer cells and tumor microenvironment (TME) cells (7–9). Cilia are essential for ovulation, ovarian development and fertility (10). Researchers who have investigated hydrogen peroxide, ionizing radiation, and chemotherapy effects in a human fallopian tube *in vitro* model had shown how the ciliated cells exhibited signs of DNA damage, and the TP53 was shown to function in altering ciliated cell differentiation (11) and participating in the transformation of EOC-associated mutations (12), but this phenomenon of DNA damage was not observed in serous cells. It may be possible that the ciliated cells and serous cells were affected by different stimulants: Ciliated cells are mainly stimulated by 17-estradiol, whereas serous cells are predominantly stimulated by progesterone (13–15). A previous study also demonstrated that the DNA damage response is one of the features of ciliary dysfunction, which is also similar to a feature of EOC (16). DNA damage in cells generally has complex repair pathways to prevent genomic instability that can lead to cancer (17). The ideal strategy to investigate this phenomenon would be to regulate cilia-associated gene expression in an *in vitro* model, and to prepare a toxic stress environment using over-drainage of follicular fluid to evaluate the DNA damage of ciliated cells (18). According to the WHO classification, there are five subtypes of ovarian cancer. High-grade serous carcinoma (HGSOC), low-grade serous carcinoma (LGSC), endometrioid carcinoma (EC), clear cell carcinoma (CCC), and mucinous carcinoma (MC) have different clinical behaviors, therapeutic responses, and prognostic outcomes. High-grade serous carcinoma is considered to be epithelial ovarian cancer derived from fallopian tube lesions. More than 90% of *HGSC* are found to have *TP53* mutations, and in nearly 50% of cases, there are underlying aberrations in *BRCA1* and *BRCA2* (19). In individuals with *BRCA* mutations, a group of tubal epithelial lesions was found. Combined with nuclear atypia, P53 mutation and Ki-67 index, a series of tubal lesions and tubal intraepithelial carcinoma (STIC) characterized by *P53* can be identified. Therefore, tubal lesions are considered to be early events of epithelial ovarian cancer (20). From a clinical perspective, a previous study reported that, compared with women in the general population, patients with ovarian cancer with *BRCA* mutations have an average reduction of 50% in the number of ciliary cells (18). In addition, the associated genetic and environmental factors involved in the pathogenesis of EOC were shown to affect the functions of tubal cilia, indicating that tubal ciliary cells may be involved in the occurrence and development of EOC (21).

To understand better the role of tubal cilia in the development of ovarian cancer, it is particularly important to conduct a comprehensive analysis of tubal cilia-associated genes. We hypothesized that oviduct ciliary cell dysfunction may increase the toxic stress environment after ovulation by affecting the clearance of follicular fluid by cilia, thereby providing a basis for the development of EOC. To date, however, no experimental data have been obtained to directly support this hypothesis. Therefore, we aimed to evaluate the relationship between fallopian tube ciliary loss in patients with EOC and the presence of the cilia-associated gene *HYDIN*, and the incidence of ovarian cancer. The resultant findings have enabled us to hypothesize that cilia-associated genes are expressed in ciliated cells and mediate disease development to provide a reference for the exploration of the influence of these genes on the prognosis of EOC.

## Materials and methods

### Samples

Surgical resection specimens were collected. The disease group was ovarian cancer tissue and fallopian tube tissue from 80 patients with ovarian cancer, and the healthy control group was prophylactic resection of ovaries and fallopian tube tissue from 50 non-cancer patients (ovarian cancer tissue samples were obtained from the ovarian site). The samples were obtained from the Sixth Affiliated Hospital of Sun Yat-sen University between January 2015 to June 2019. The studies involving human participants were reviewed and approved by the Ethics Committee of the Sixth Affiliated Hospital of Sun Yat-sen University. Patients had signed consent at the time of admission to support the use of pathological specimens for clinical research.

The inclusion criteria for the patients were as follows: i) from January 2015 to the present, the patients(with preserved stage I-IV primary ovarian cancer that had been pathologically confirmed) had undergone surgical resection with medical intent, with formalin-fixed paraffin-embedded (FFPE) cancer tissue and corresponding fallopian tube samples; ii) the patients with ovarian cancer were under the age of 75; iii) at least 10 samples of FFPE tissue sections should ideally be provided, and the surface area of the ovarian tumor in paraffin sections should ideally be>25%; iv) the fallopian tubes and ovaries in the control group were prophylactically excised by hysterectomy in non-cancer patients, and no abnormalities such as pathological changes and inflammation were confirmed by the final pathological results; v) all the study subjects had normal ovulatory function without history of ovulation-promoting therapy prior to inclusion; vi) all the included study subjects had no history of tubal or ovarian inflammation. The exclusion criteria were as follows: i) patients who had received preoperative treatment, including radiotherapy, chemotherapy, targeted therapy, and immunotherapy, on account of avoiding the adaptive evolution of tumor genomes caused by treatments that may have affected the statistical results; ii) missing or insufficient tissue mass, or the tumor surface area of the paraffin section was <25%, or the samples were of a quality that did not meet the requirements of subsequent experiments (such as sample degradation); iii) samples with incomplete clinical information (such as tumor type, clinical classification stage, etc.); iv) samples of non-primary ovarian malignancy. In our study, all the patients who underwent prophylactic hysterectomy were not tested for BRCA mutations because they had no pathological predisposition to cancer.

### Hematoxylin and eosin staining

The first continuous section of each patient sample was subjected to H&E staining. A histological examination was performed for each H&E section. A Leica DSM 2500 optical microscope (Leica Microsystems, Inc.) was used to observe the morphology of each group of tubal epithelial cells. Simultaneously, ten high-magnification (400-fold) microscopic fields were randomly selected for each sample to manually count the numbers and ratio of the ciliated cells (cf. non-ciliated cells). The frequency of cilia was determined by dividing the number of cilia cells by the total number of nuclei. The position of the fallopian tube cilia was marked in the 400-fold microscopic images for subsequent identification and analysis using an immunohistochemical (IHC) method, as described below.

### IHC analysis

After dewaxing and rehydration, the sections were immersed in 0.3%H_2_O_2_ at room temperature for 15 min, the endogenous peroxidase activity was inactivated, the slides were rinsed with PBS (3 washes, 5 min each wash), the tissues were blocked with 2.5% normal serum (Beijing Pulilai Company, catalogue number C1779) for 10 min, and subsequently the sections were incubated with primary anti-HYDIN antibody (dilution, 1:100;anti-Cat IgG; Dako, catalogue number24741-1-AP) and Epitomics^®^anti-acetylated α-tubulin (1:200; anti-rabbitIgG; Abcam). The antibodies were diluted in the antibody dilution buffer solution at 4°C overnight. After washing with PBS (3 washes, 5 min each wash), horseradish peroxidase (HRP)-conjugated secondary antibodies (Beijing Pulilai Company, catalogue number:abc20023, 1:500) at 37°C were incubated with the tissue sections at room temperature for 30 min. Subsequently, the tissue sections were washed with PBS (3 washes, 10 min each wash). Then, DAB (diamine oxide benzidine) color development was performed (each section was added with 1 drop of fresh DAB color development agent). After color development, the reaction was terminated immediately by placing in flowing water, and the color development time did not exceed 3 minutes. Hematoxylin counterstained: Soak in hematoxylin solution for 1 min and in running water for 5 min. Differentiation and return to blue: differentiation in 1% hydrochloric alcohol for 3 seconds, and return to blue for 10 min with running water. Dehydration: the sections were successively immersed in the following reagents, 85%, 95%, 100% and 100% ethanol for 2 minutes each; An additional 2 minutes of xylene transparency. For sealing: a drop of neutral gum was placed in the center of the tissue, the cover glass was pressed against the tissue, the tissue was left to dry at room temperature, and labeled. Finally, the staining results of tissue sections were observed under a light microscope, photographed under different magnifications, and recorded. The histochemical staining results were then assessed according to the positive or negative staining of the sections. Software counting and scale-bar tools (Leica Microsystems, M80, Inc.) were used for the measurements. The expression of acetylated tubulin in cilia in at least 10 of the 400-fold microscopic images was evaluated by counting for each tissue type, and the cilia were counted manually. The frequency of cilia was determined by dividing the number of cilia cells by the total number of nuclei. Cells at the edge of the image were excluded, and no threshold was applied to the analysis to maintain the consistency of the scores among samples. The IHC scoring criteria were as follows. For α-tubulin, the positive cells were defined by the expression of α-tubulin in ciliary cells. Five high-power fields were randomly selected, and the percentages of positive cells were counted. Positive staining was defined when >25% of cells were observed to express α-tubulin; negative staining was defined when the number of cells expressing α-tubulin was <25%. For *HYDIN*, using a semi-quantitative scoring method (i.e., the positive cell number x degree of positivity), a score >1 was considered to be positive, and the degree of positivity was defined as 0 or 1according to whether *HYDIN* had a tan reaction in the tubal ciliary cells and ovarian cancer cells. Ten high-power fields were randomly selected, and a score of 0 was defined by the percentage of positive cells being below 25%, whereas a score of 1 was defined when the percentage of positive cells was 25%-50%, and a score of 2 was defined when the percentage of positive cells was ≥50%. When the score of positive cell number x degree of positivity was 0, it was defined as negative, and was defined as positive was defined with the score of positive cell number x degree of positivity ≥1.

### Observation of the morphology of the cilia under a transmission electron microscope

10 cases of EOC patients and healthy groups were selected to observed under electron microscope, the tissues described above were rinsed with PBS (pH=7.2) and subsequently fixed in 2% glutaraldehyde (MilliporeSigma) for 1 hour at 4°C. The tissues were then rinsed 3 times with PBS for 5min, fixed with 1% osmium acid (2 h at 4°C), and then rinsed again (3 times with PBS). The sections were dehydrated using a step-by-step ascending ethanol series, acetone was soaked with resin, and the sections were embedded in epoxy-resin. After slicing the sections using an LKB V ultra-thin slicer, they were dyed using uranium acetate and lead citrate. Finally, the images were observed with a Philips CM-120 transmission electron microscope (Philips Medical Systems, Inc.).

### Whole-exome sequencing

67 samples with cilia loss were sent to the TopGene Clinical Diagnostic Laboratory (Zhongshan, China) for WES (Original data generated using Whole exome sequencing had submitted to a public database: https://ngdc.cncb.ac.cn/search/?dbId=hra&q=HRA000294&page=1). Genomic DNA extraction was performed using a Mag-Bind^®^ blood and tissue DNA HDQ 96 kit (Omega Bio-Tek, Inc.) according to the manufacturer’s instructions. A UV spectrophotometer (NanoDrop Technologies; Thermo Fisher Scientific, Inc.) was used to check the DNA quality. DNA quantification was performed using a Qubit™ 3 Fluorometer (Thermo Fisher Scientific, Inc.). Exome capture from the genomic DNA was performed using the WES-T086V5 panel (NingBo iGeneTec Technology Co., Ltd.). PCR products were checked for their quality using a LabChip^®^ GX Touch™ 24 Nucleic Acid Analyzer (PerkinElmer, Inc.). Pair-end sequencing was performed using an MGISEQ-2000 sequencer (MGI Tech Co. Ltd.) according to the manufacturer’s instructions. The average depth of each sample was 100X, and the read length was 150bp.

### Reverse transcription-quantitative PCR analysis

Four experimental treatment groups [epithelial ovarian cancer tissue (EOC group, n=30), tubal tissue (cilia in EOC group, n=30), normal ovarian tissue (normal group, n=15), and tubal (normal fallopian tube (NTC group, n=15))] were created for the detection of *HYDIN* mRNA. After the above tissues had been grounded, total RNA was extracted using Invitrogen^®^TRIzol™ (Thermo Fisher Scientific, Inc.). Following the instructions of the reverse transcription kit of Takara Biotechnology Co., Ltd., the reaction system comprised a total volume of 20 µl: SYBR^®^ Green mix, 10 µl; upper and lower primer mixture, 0.5 µl each; cDNA, 5 µl; and ddH2O, 4.0 µl. The thermocycling reaction conditions were as follows: Pre-denaturation at 50°C for 20 seconds, followed by denaturation at 95°C for 5 minutes, and annealing extension at 60°C for 34 seconds (for a total of 39 cycles). Analysis of the dissolution curve was performed at a temperature range of 60-95°C, and the transcripts were read once per minute. The primer sequences were as follows: *HYDIN* upstream sequence:5’-TCCTTTGGGTTTCCTCATACC-3’, and downstream sequence:5’-TAGTCCACATGCTGCTCACA-3’. *GAPDH* upstream sequence:5’-ACAACTTTGGTATCGTGGAAGG-3’, and downstream sequence:5’-GCCATCACGCCACAGTTTC-3’. According to the Cq value, the experimental data were quantified using the 2^-ΔΔCq^ method.

### Western blot analysis

Four experimental treatment groups [epithelial ovarian cancer tissue (EOC group, n=30), tubal tissue (cilia in EOC group, n=30), normal ovarian tissue (normal group, n=15), and tubal (normal fallopian tube (NTC group, n=15))] were extracted from fresh tissues using a nuclear extraction kit (KaiJiKGP150). Protein concentration was determined by Bradford colorimetric assay. The protein supernatant was mixed with 5× SDS loading buffer and denatured at 98°C for 10 min. 20 ul of the denatured protein sample and 5 ul of marker were added to the loading gel Wells, and the electrophoresis program was 80 V for 20 min and 120 V for 70 min. After electrophoresis, the membrane was transferred to PVDF membrane for 70 minutes using a constant current (200 mA). After 2 hours of incubation in 5% skim milk powder at room temperature, the membranes were washed three times with PBST at 5 min intervals, and then anti-*HYDIN* primary antibody (1:1,000, Dako; Agilent Technologies, Inc. catalogue number 24741-1-AP) was added for incubation at 4°C overnight. After incubation with the primary antibody, the membranes were washed five times with PBST at 5 min intervals, and the secondary antibody of horseradish peroxidase conjugate (1:2500, Sheep rabbit IgG/HRP, BOSTER Biological Technology Co. catalogue number: YJ0189) was added. Sheep rabbit IgG/HRP, BOSTER Biotech, YJ0189). The internal reference antibody was β-actin (1:1000, Abcam, catalog number: ab8227). ECL luminescence solution (Kai Ji Biological Technology Co, 1ml) was used for luminescence imaging photography. ImageJ software (V1.8.0) was used to analyze the intensity of each antibody. Relative gray value for each protein = gray value/internal reference.

### Statistical analysis

SPSS20.0 (IBM Corp.) and GraphPad statistical software (GraphPad Software, Inc.) were used for data analysis. The data of each group were expressed as the mean ± SEM. The student’s t-test was used for analyzing statistical difference between two groups, and the Bonferroni test performed following one-way ANOVA was used for comparison between three or more groups. P<0.05 was considered to indicate a statistically significant difference.

## Results

### Baseline characteristics

The histological details of the 80 cases of ovarian cancer were as follows: 47 cases of high-grade ovarian cancer, 14 cases of low-grade ovarian cancer, 8 cases of clear cell carcinoma, 3 cases of mucinous carcinoma, and 8 cases of other carcinoma (**Table 1**). Details of the clinical cases of the samples were as follows: Stage: I: 19 cases; stage II: 7 cases; stage III: 35 cases; stage IV: 15 cases; and undetermined: 4 cases, whereas of the 80 samples, 49 cases were poorly differentiated, 9 cases were moderately differentiated, 5 cases were highly differentiated and 17 cases were not determined. The average age of the 80 patients with ovarian cancer was 52.33 ± 10.26 years old (45~65 years old, Median: 53.4 years old), whereas the average age of the 50 non-ovarian cancer patients was 50.98 ± 7.15 years old (43~65 years old, Median:51.5 years old). There were no significant differences of Cilia loss in ages (<45 and >45, P = 0.4438), stages (I-II and III-IV-Undetermined, P = 0.9652), and differentiation (Low-Medium and High-Undetermined, P = 0.4412) (**Table 2**).

**Table 1:** Clinicopathological features of patients with epithelial ovarian cancer (EOC) and EOC with cilia loss in the fallopian tubes.

	EOC (n=80)	Histotype					Cilia loss (n=67)
		High-grade serous (n=44)	Low-grade serous (n=11)	Clear cell carcinoma (n=10)	Mucinous carcinoma (n=8)	Other (n=7)	
Age at diagnosis (years)							
Mean ± SD	52.33 ± 10.26	52.3 ± 8.0	50.2 ± 12.4	47.5 ± 12.1	42.0 ± 13.1	47.9 ± 11.0	51.12 ± 8.54
Age <45	34	20	7	5	2	0	30 (44.8%)
Age >45	46	24	4	5	6	7	37 (55.2%)
Stages							
I	19	11	2	1	2	3	17 (25.4%)
II	7	2	3	1	1	0	5 (7.5%)
III	35	24	6	2	2	1	32 (47.8%)
IV	15	6	0	5	2	2	12 (17.9%)
Undetermined	4	1	0	1	1	1	1 (1.5%)
Differentiation							
Low	49	31	5	5	4	4	44 (65.7%)
Medium	9	4	3	0	2	0	6 (9.0%)
High	5	2	1	2	0	0	3 (4.5%)
Undetermined	17	7	2	3	2	3	14 (20.9%)

SD, standard deviation; EOC, epithelial ovarian cancer.

**Table 2:** Significance between epithelial ovarian cancer (EOC) and EOC with cilia loss in the fallopian tubes.

	Stages		Differentiation		Age	
	I-II	III-IV-Undetermined	Low-Medium	High-Undetermined	<45	>45
**EOC (n=80)**	26	54	58	22	34	46
**Cilia loss (n=67)**	22	45	50	17	30	37
**P**	0.9652		0.7712		0.0987	

EOC, epithelial ovarian cancer; NTC, normal fallopian tube of control.

### Observation of the loss of tubal cilia as determined by H&E staining and IHC analysis

**Figure 1:**
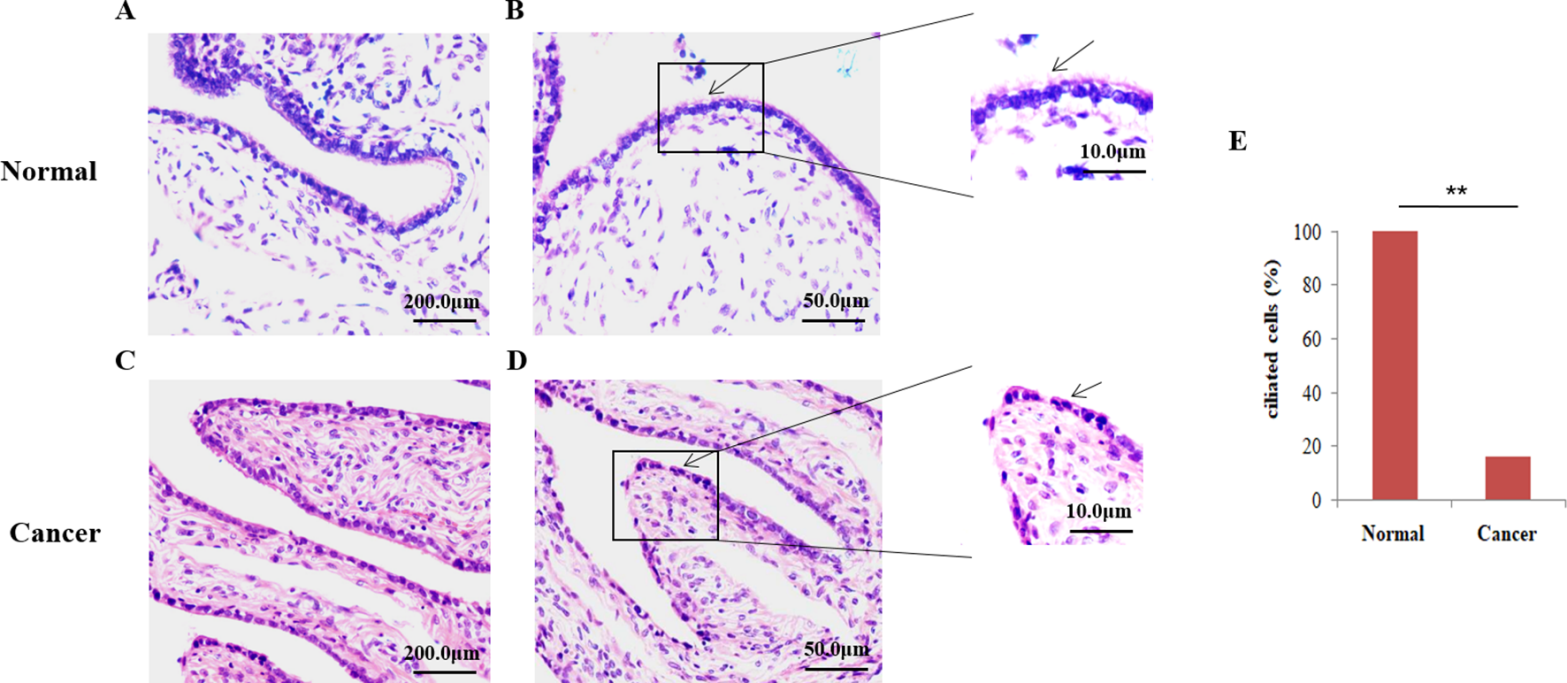
Microscopic analysis of the ciliate by hematoxylin and eosin (H&E) staining. **(A, B)** H&E staining was employed to show that cilia are easily identified on the fallopian tubes of control normal human tissue. **(C, D)** Fallopian tubes cilia (FTC) were not easily observed in the epithelial ovarian cancer (EOC) tissue., the arrows show the detection of cilia using H&E staining. **(E)** The percentage of ciliated cells between normal tissue and EOC. **P<0.001.

To assess the loss of fallopian tube cilia in patients with ovarian cancer, fallopian tube samples from 80 patients with EOC and 50 healthy ovaries were stained with H&E staining. Based on these experiments, distinct cilia were identified on the oviduct of the healthy control group (**Figures 1A, B**), whereas the tubal cilia in the ovarian cancer group were significantly reduced (**Figures 1C, D**). The abnormal rate of cilia was subsequently calculated. Only 16.25% of the samples were found to be ciliated in EOC, and the percentage of cilia in 67 samples was significantly reduced (P=0.000), whereas all the fallopian tubes exhibited cilia in the healthy control group (**Figure 1E**).

**Figure 2:**
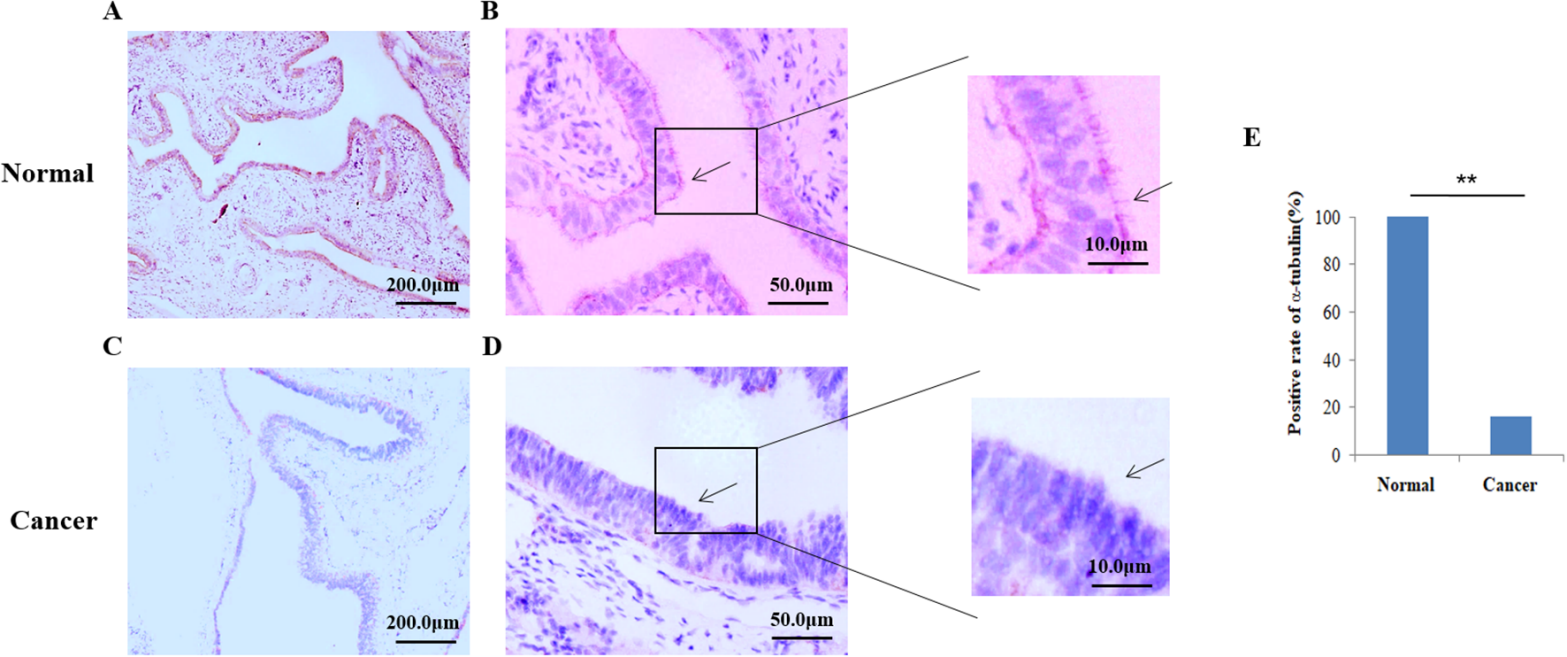
Expression of acetylated α-tubulin in the tubal tissue. Immunohistochemistry (IHC) was used to detect ciliary markers; acetylated α-tubulins were shown in pink. **(A, B)** Cilia were easily located on the fallopian tubes of control normal human tissue However, in the ovarian group **(C, D)**, the cilia were significantly reduced. The arrows show cilia detected using the IHC method. **(E)** The Positive rate of acetylated α−tubulin between normal humans and patients with epithelial ovarian cancer. **P<0.001.

Subsequently, the ac-tubulin was detected in the fallopian tube epithelium in 100% of healthy samples, and in 16.25% EOC samples. (cilia shown in **Figures 2A, B**), while the percentage of positive tubal ciliary markers was significantly reduced in the EOC group (16.25%; P<0.001) (**Figures 2C–E**, **Table 3**).

**Table 3:** Expression of α-tubulin in EOC patients and healthy group.

Group	Cases	Number of cells expressing α-tubulin (n/%)			
		Number 0-10%	Number 11-24%	Number 25-49%	Number ≥50%
**Healthy**	50	0 (0)	0 (0)	12 (24.00)	38 (76.00)
**EOC**	80	3 (3.75)	10 (12.50)	55 (68.75)	12 (15.00)
**P**		0.000			

EOC, epithelial ovarian cancer; NTC, normal fallopian tube of control.

**Figure 3:**
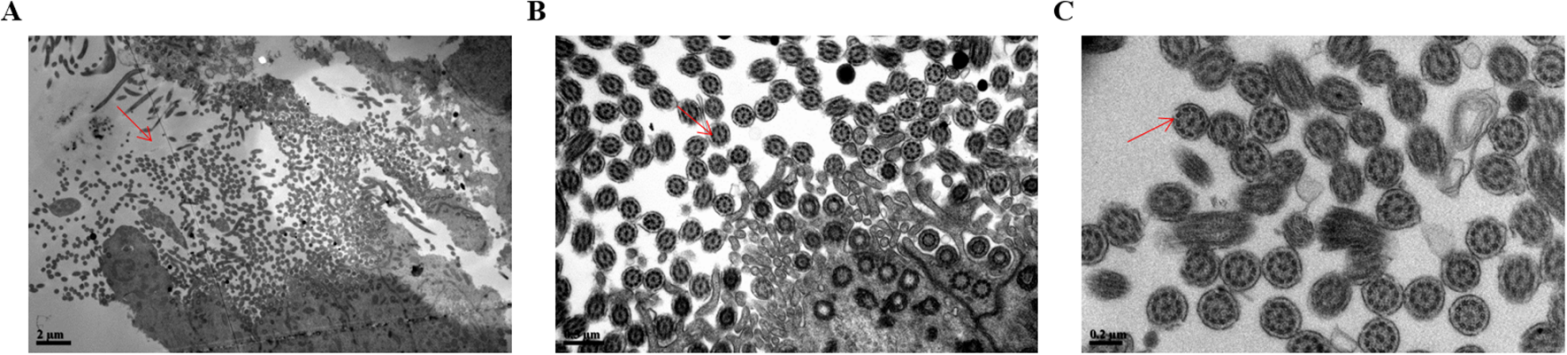
Scanning electron microscopy analysis of the surface in normal fallopian tube cilia tissue. **(A)** The ciliary 9 + 2 structure in magnifications of bar=2µm was shown by the arrow. **(B)** The ciliary 9 + 2 structure in magnifications of bar=0.5µm was shown by the arrow. **(C)** The ciliary 9 + 2 structure in magnifications of bar=0.2µm was shown by the arrow. The arrow showed the cilia 9 + 2 structure, namely a pair of central microtubules and 9 peripheral microtubules).

**Figure 4:**
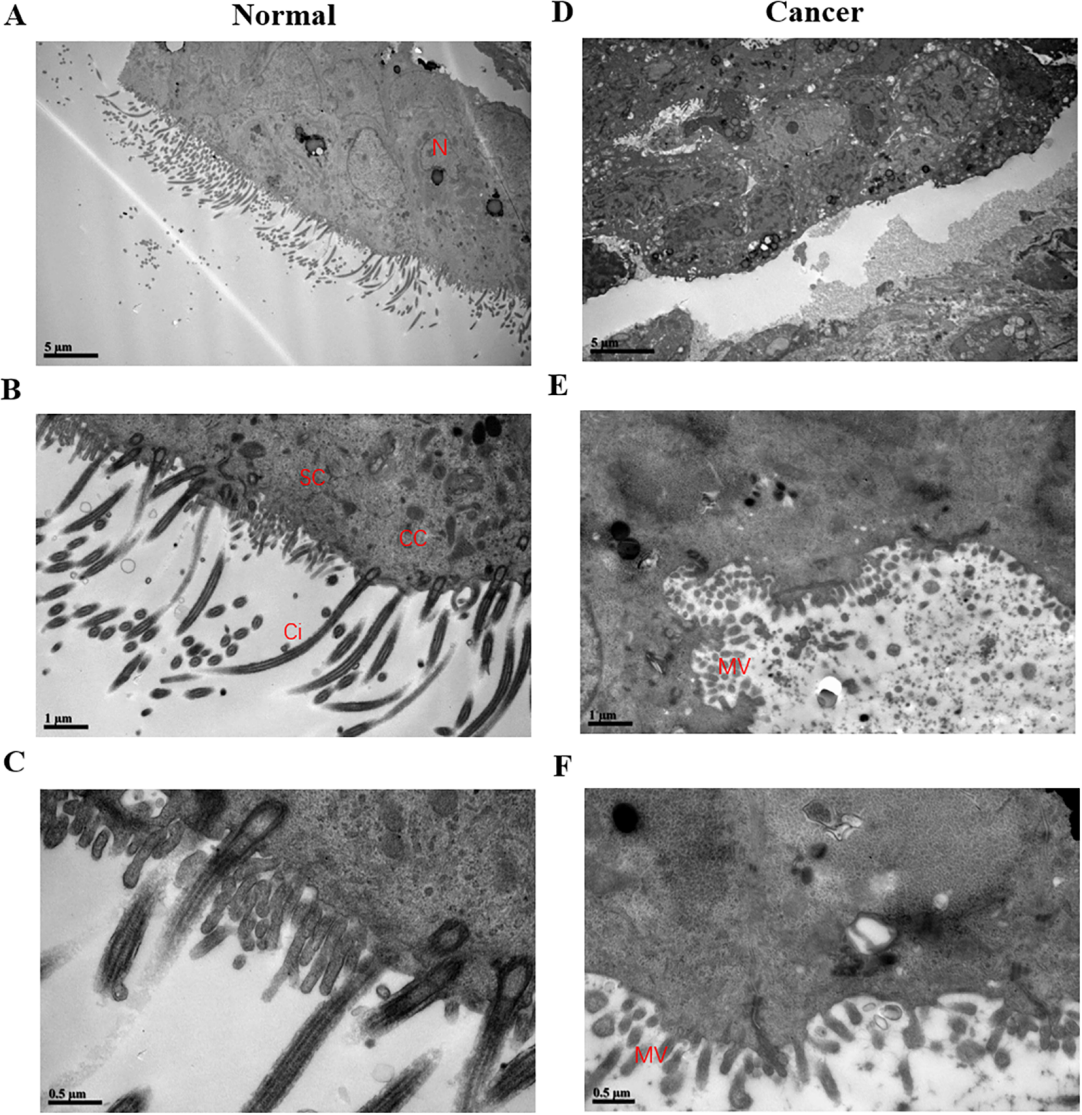
Images of fallopian tubes in the normal control group and the ovarian cancer group detected by scanning electron microscopy. **(A–C)** Images of the normal control group were presented, showing the presence of numerous cilia. **(D–F)** Images of the ovarian cancer group were shown, revealing the presence of only a few ciliated structures. **(A, D)** Bar=5 µm; **(B, E)** bar=1 µm; **(C, F)** bar=0.5µm. The labels in the figure denoted the following, detected with scanning electron microscopy. N, nucleus; SC, secretory cell; CC, fibrinoid cell; Ci, cilia; MV, microvillus.

In addition, to confirm ciliary loss in the tubal samples in patients with ovarian cancer, the fallopian tube cilia tissue of the healthy control group and patients with ovarian cancer were observed and compared using a scanning electron microscope. The electron microscopy experiments showed that the cilia in the healthy control group were neatly arranged, where the cilia cells showed low electron density, and the typical “9 + 2” microtubule structure was clearly visible. The cilia had a central axon, which was composed of 9 peripheral microtubule pairs (“9 + 2” structure, as shown by the arrows in **Figures 3A–C**). The secretory cells have short microvilli on the surface, and apical secretion is one of their functions (**Figures 4A–C**). Nevertheless, the cilia were associated with degenerative phenomena, such as cilia shedding, shortening, and microtubular edema, in patients with ovarian cancer. In these cases, the number of cilia was markedly reduced, and the typical cilia “9 + 2” structure was not observed (**Figures 4D–F**).

### The loss of tubal cilia in ovarian cancer is associated with reduced expression levels of genes required for cilia development

**Figure 5:**
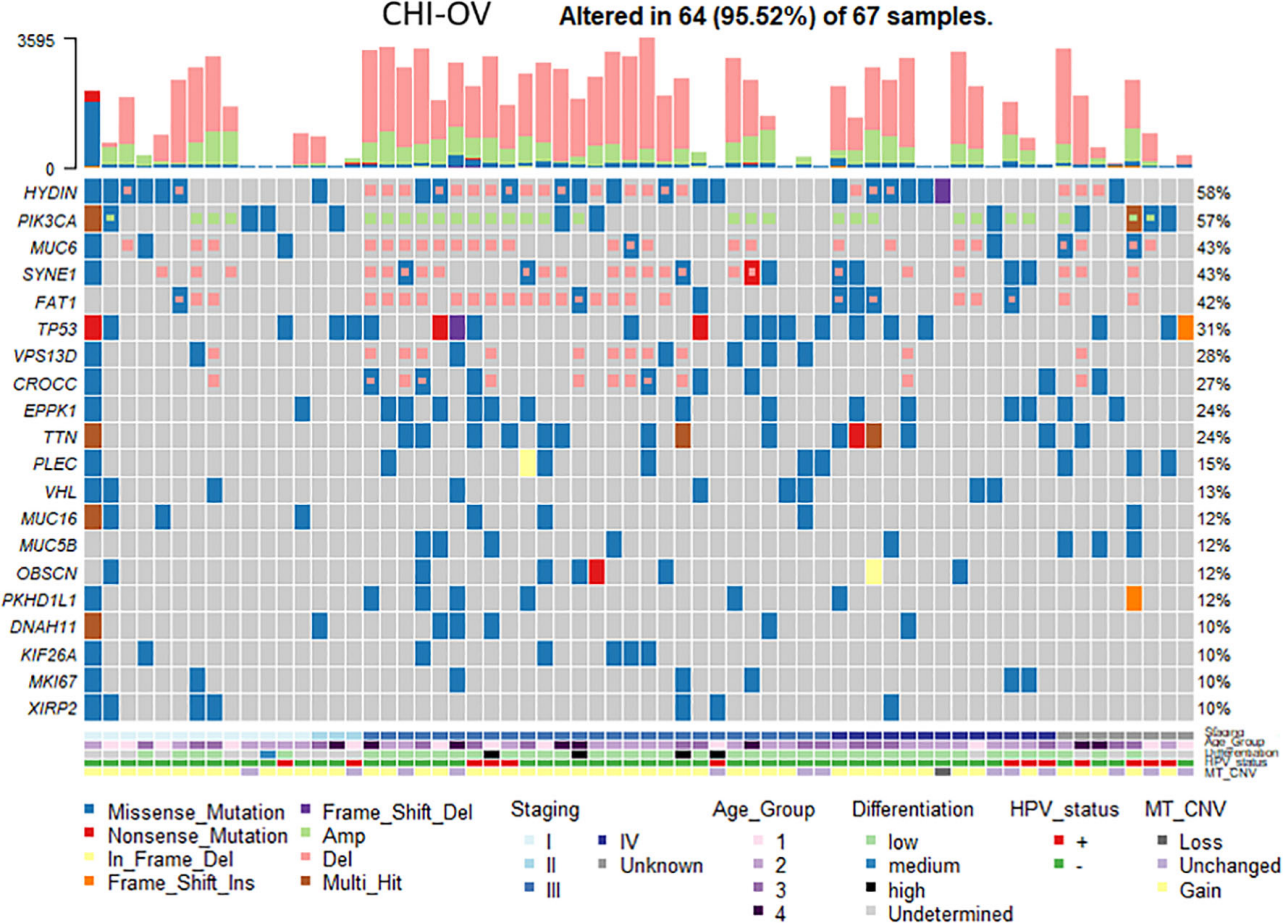
Analysis of the genetic mutations in ovarian cancer samples with ciliated loss. A heat map revealed the changes of cilia-associated gene in ovarian cancer tissue samples with different clinicopathological features. Each column represents a sample of ovarian cancer tissue, and each row represents the relative expression of different ciliary genes (the gene names are shown on the left). Different colors represent different clinicopathological features.

The loss of tubal cilia in ovarian cancer is associated with reduced expression levels of genes required for cilia development. In order to determine whether cilia-related genes are altered when fallopian tube cilia are missing in ovarian cancer patients, we screened the gene mutation spectrum in ovarian cancer (**Figure 5**). We found high frequency mutations in 20 ciliates-related genes, including *HYDIN, PIK3CA*, *MUC6*, *PEDE4DIP*, *SYNE1*, *FAT1*, *HLA-AQA2*, and *TP53*. *HYDIN* is a gene closely related to tubal cilia. Featured at the top of the list of frequently mutated genes (mutated in 58% of the samples) was the *HYDIN* gene, which encodes a central pairing protein associated with C2 microtubules. The figure also shows these ovarian cancer patients in different age groups (age <45, group 1; 45≤ age <55, group 2; 55≤ age <65, group 3; age ≥65, group 4), Tumor Node Metastasis (TNM) stage, and tumor cell differentiation status.

### Abnormal expression of HYDIN in EOC and its association with tubal cilia loss

**Figure 6:**
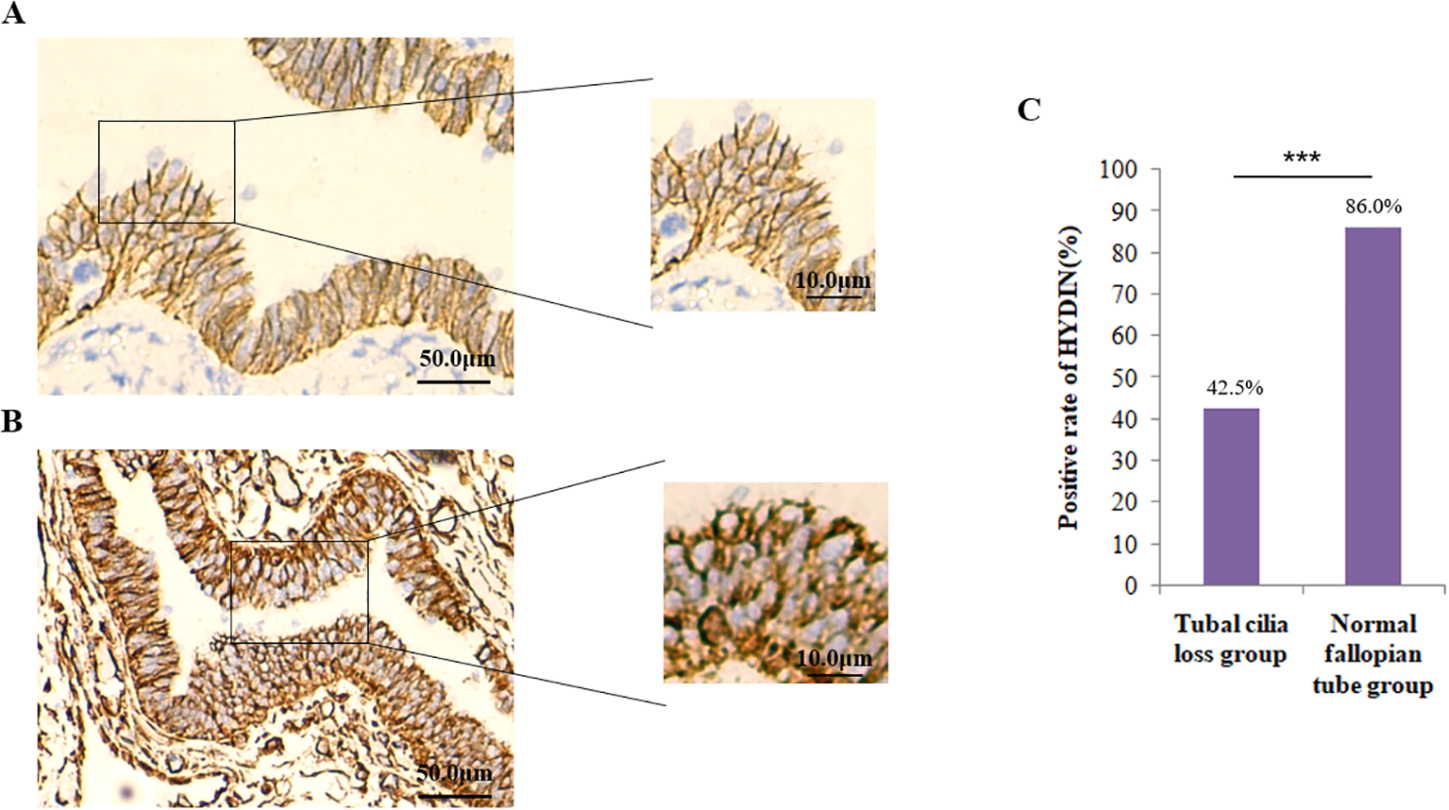
Expression of HYDIN protein in oviduct tissues detected by the immunohistochemical (IHC) method. **(A)** Expression of HYDIN protein on fallopian tubes in EOC group. The HYDIN protein positive reactions are shown by the brown coloration in tubal epithelial cells. **(B)** HYDIN protein expression in the fallopian tubes of normal control group. **(C)** The positive rate of tubal HYDIN protein in EOC group and normal control group. ***P<0.0001.

Firstly, the IHC results showed that the positive expression rate of *HYDIN* protein in the tube tissues of EOC group (42.5%) was significantly lower than that of the Healthy control group (86.0%) (P=0.000) (**Figure 6**, **Table 4**).

**Table 4:** Expression of HYDIN in oviduct tissues in EOC group and healthy group.

Group	Cases	HYDIN expression (n/%)		
		Scores 0	Scores 1	Scores 2
**Healthy**	50	7 (14.00)	10 (20.00)	33 (66.00)
**EOC**	80	46 (57.5)	19 (23.75)	15 (18.75)
**P**		0.000		

EOC, epithelial ovarian cancer; NTC, normal fallopian tube of control.

**Figure 7:**
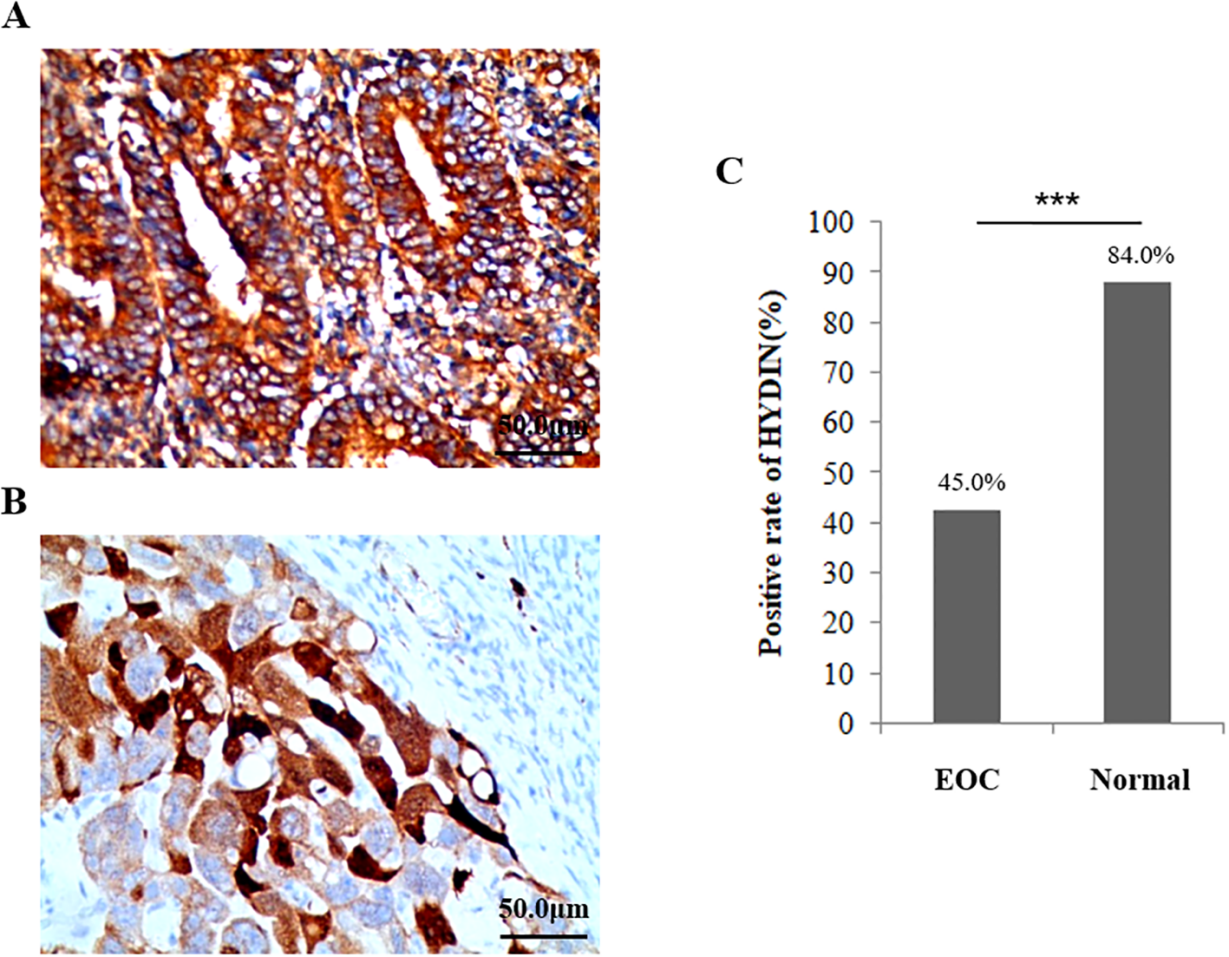
Expression of HYDIN protein in ovarian cancer tissue and the normal ovarian group detected using the IHC method. **(A)** Expression of HYDIN protein in ovarian cancer tissues of EOC group. **(B)** Expression of HYDIN protein in ovarian tissue of normal control group. The HYDIN protein positive reaction is shown by the brown coloration in ovarian cancer cells. **(C)** The positive rate of HYDIN protein between ovarian cancer tissue in EOC group and ovarian tissue in normal control group. ***P<0.0001.

The IHC results showed that the expression level of *HYDIN* protein was significantly different in ovarian tissues of ovarian cancer group (45.0%) and Healthy control group (84.0%)(P=0.000) (**Figure 7**, **Table 5**). The trend of *HYDIN* positive expression in ovarian cancer and fallopian tube tissues in EOC group was significantly lower than that in control group. This indicated that the loss of tubal cilia in EOC group was accompanied by decreased expression of cilia-related gene *HYDIN*.

**Table 5:** Expression of HYDIN in OC tissues in EOC group and healthy group.

Group	Cases	HYDIN expression (n/%)		
		Scores 0	Scores 1	Scores 2
**Healthy**	50	8 (16.00)	13 (26.00)	29 (58.00)
**EOC**	80	44 (55.00)	21 (26.25)	15(18.75)
**P**		0.000		

EOC, epithelial ovarian cancer; NTC, normal fallopian tube of control.

**Figure 8:**
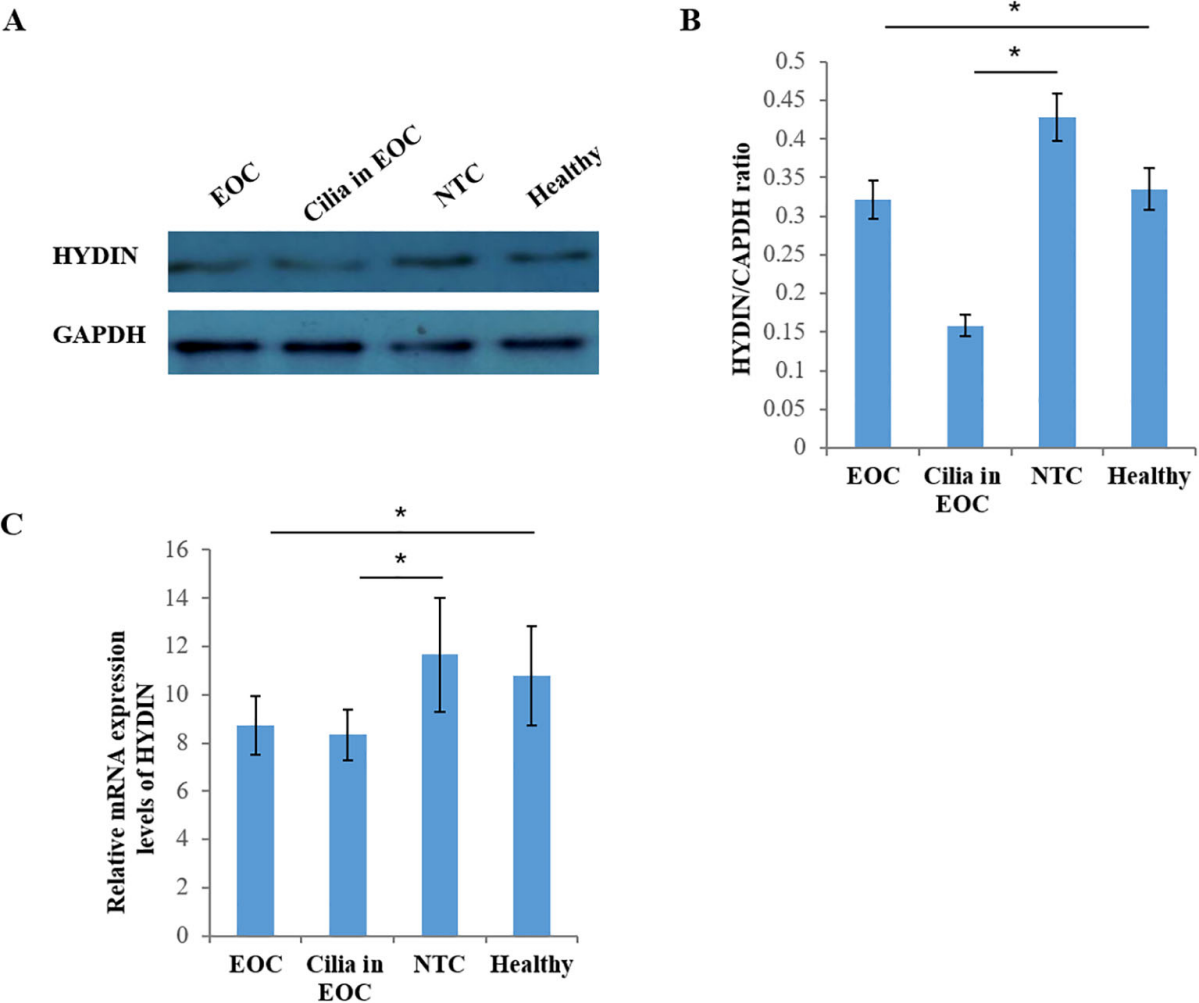
Expression of HYDIN protein in ovarian cancer and fallopian tube tissue by Western blot. **(A)** Expression of HYDIN protein in ovarian cancer tissue (EOC), oviduct tissue (Cilia in EOC), oviduct tissue (NTC) of normal control group and ovarian tissue (normal) of Healthy control group. **(B)** HYDIN/GAPDH ratio of EOC, Cilia in EOC, and NTC, and Healthy groups. **(C)** Reverse transcription-quantitative (RT-q) PCR was used to detect HYDIN-mRNA in EOC, Cilia in EOC, and NTC, and Normal groups. *P<0.05.

Thirdly, the western blotting results showed the expression level of HYDIN protein in ovarian cancer and fallopian tube tissues in EOC group was significantly lower than that in the Healthy control group, and these differences reached the level of statistical significance (P<0.05) (**Figures 8A, B**, **Table 6**). Similarly, the RT-qPCR analysis also revealed differences in the expression levels of *HYDIN* mRNA between the ovarian cancer groups and the Healthy control groups (P<0.05) (**Figure 8C**, **Table 6**).

**Table 6:** Expression of HYDIN in OC tissues in EOC group and Healthy group by Western blot and PCR.

Group	Cases	HYDIN expression	
		Western blot	PCR
**EOC**	30	0.321 ± 0.025	8.741 ± 1.225
**Cilia in EOC**	30	0.158 ± 0.014	8.338 ± 1.058
**NTC**	15	0.428 ± 0.031	11.658 ± 2.365
**Healthy**	15	0.335 ± 0.027	10.782 ± 2.051
**P**		0.000	0.000

EOC, epithelial ovarian cancer; NTC, normal fallopian tube of control.

## Discussion

Epithelial ovarian cancer (EOC) is one of the three major gynecological cancers. The global incidence of ovarian cancer accounts for 1.6% of all cancers, with 207,252 deaths per year, the mortality rate is high, and the 5-year survival rate is extremely low, with the overall survival rate of ovarian cancer stage III only 40%, and stage IV as low as 20% (22). The mortality of ovarian cancer is decreasing with the progress of surgery and chemotherapy in recent years, EOC in the advanced stages does not respond to conventional chemotherapy efficiently (23, 24). At present, the routine screening method of ovarian cancer is mainly based on serum CA-125 detection combined with the results of vaginal color Doppler ultrasound. However, some studies have shown that the sensitivity of CA125 in early ovarian cancer is low, and the sensitivity of stage I is as low as 25% (25). Routine screening of ovarian cancer by detection of serum CA-125 and vaginal color Doppler ultrasound alone does not reduce the mortality of ovarian cancer, but increases the probability of unnecessary surgery (26, 27). To improve the treatment prospects for patients, it is necessary to intensify studies on the tumor biology of ovarian cancer. With the progress of research, it has been found that there are two types of ovarian cancer initiation cells: fallopian tube epithelial (FTE) cells and ovarian surface epithelial cells (OSE), both of which are developed from the mesoderm, and the anatomical position of the two is very close. Researchers have shifted the focus of EOC origin from ovarian surface epithelial cells to fallopian tube epithelial cells (28–30). Interestingly, it has been found that stem cells periodically reproduce in the oviduct umbrella, where Mullerian and coelomic epithelial cells fuse, and the majority of serous tubal intraepithelial lesions (STIL) occur in the oviduct umbrella (31). Additionally, as EOC is a diverse tumor, the cells of origin are unclear. Fathalla has proposed the “continuous ovulation” hypothesis: continuous ovulation cycles in women during their reproductive years increase a woman’s risk of EOC, ovulation leads to increased inflammation, and DNA damage can be induced in the ovarian cortex for some reason, such as the secretion of certain cytokines, chemokines, and hormones. These processes can lead to the development of tumors (32, 33). The ovarian oviposit lifetime ovulation number has been shown to be positively correlated with an increased risk of ovarian cancer occurrence, although, on the other hand, using the contraceptive pill to prevent ovulation may proportionately reduce the risk of ovarian cancer (33). However, the biological mechanism that would explain the association between the lifetime ovulation number and the risk of EOC remains controversial. Some scholars considered that continuous ovulation would damage the surface epithelium of the ovary; over time, the surface epithelium of the ovary would be repeatedly damaged and repaired, thereby providing suitable grounds for the occurrence of ovarian cancer (34). However, another research group proposed that the mechanism associated with the risk of EOC was that the oviduct epithelial cells were repeatedly stimulated by hormones during the normal menstrual cycle, which may also explain the increased risk of EOC in patients with menopausal hormone replacement therapy, where a positive correlation has been demonstrated (35). Certain researchers have also hypothesized that the timing of ovulation would release inflammatory mediators and recruit inflammatory cells, causing inflammatory damage and oxidative stress to the tubal epithelium. With the passage of time, such damage would occur repeatedly in the tubal region, and this would possibly induce the occurrence of ovarian cancer (36, 37). Perets et al. induced mutations in *BRCA*, *TP53*, and *PTEN* in fallopian tube secretory epithelial cells (FTSEC) and found that this led to the development of EOC, which subsequently developed into ovarian cancer and peritoneal metastasis (38). However, whether the loss of tube cilia is involved in the above events remains unclear. Although studies have shown that tubal ligation (tubal removal) can reduce the risk of ovarian cancer in high-risk individuals with *BRCA* mutations and in the general population (39). A meta-analysis of 13 cohort studies showed a 34% reduction in overall risk of epithelial ovarian cancer after tubal ligation (RR 0. 66, 95% CI 0. 6 ~ 0. 73). Similar associations exist among *BRCA* mutation carriers (RR 0. 68, 95% CI 0. 61 ~ 0. 75), but the role of cilia in the development of EOC remains poorly understood (40). In a previously published study, the cilia were analyzed in a small number of ovarian cancer tissue samples: In the first study, cilia were lost in 8 out of 9 samples, whereas in a second study of 11 cases, all the samples lost their cilia (41). The primary cilia have two central microtubules in the center of the lumen, surrounded by nine sets of double microtubules, and some active cilia without central microtubules connected by radial spokes, such as the cilia on the surface of the embryonic node. The close relationship between primary cilia and cancer growth is becoming an interesting topic. The tissue-specific structure of primary cilia may exist in a variety of tissues and tumors, and it is dichotomous in different tumors, which has aroused great interest of researchers (42, 43). To investigate the relationship between fallopian tube cilia loss and ovarian cancer, in this study, we used IHC analysis to investigate the fallopian tubes of non-ovarian cancer patients and ovarian cancer patients. The results showed that the positive rate of α-tubulin in normal fallopian tubes was 100%, whereas the positive rate of α-tubulin in the fallopian tube cilia of patients with ovarian cancer was significantly reduced. H&E staining also revealed clearly ciliated structures in the 50 normal fallopian tubes, whereas cilia were only observed in 16.25% of the patients with ovarian cancer. Furthermore, electron microscopy was employed to further confirm these conclusions. In the present study, we also analyzed ovarian cancer cases including all four degrees of differentiation, showing that cilia loss was the highest in samples with low differentiation (89%), increasing with the stage of differentiation. In a thyroid cancer model, primary cilia are involved in the process of apoptosis. Studies have demonstrated the role of primary cilia in sensing extracellular signaling and maintaining cell homeostasis, and the loss of cilia increases the risk of cancer (44). In certain types of cancers in humans, including renal cell carcinoma, cholangiocarcinoma, melanoma, ovarian cancer, and prostate cancer, the occurrence of widespread cilia loss has been shown (45–48). These studies have shown that the loss of cilia may promote the occurrence and development of cancer in certain parts of the human body.

It was found that the loss of cilia led to the inactivation of ciliated transcription suppressor *GLIS2*, which promoted the development of the mammary gland. In a mouse model of breast cancer, Ift88‐depleted inhibition of primary cilia formation in epithelial cells to stimulate HH signaling and promote tumorigenesis and malignant development (49–51). We hypothesized that loss of tubal cilia is an early event that promotes the development of ovarian cancer and may fulfill a synergistic role with other carcinogenic events to promote the development of ovarian cancer. It is well established that, after ovulation, follicles produce a toxic stress response in the tubal epithelial cells, and ciliated cells are not only conducive to promoting the transport of eggs through the fallopian tube to the uterine cavity, but also have a role in clearing the follicular fluid and eliminating the toxic substances after ovulation. It is worth noting that circumstantial evidence exists to suggest that the frequency of cilia oscillation is associated with risk factors of EOC, including endometriosis, which can reduce the motility of the tubal cilia (52). Hedgehog signaling promotes tumor growth by acting as a carcinogen that alters TME. Abnormal activation of Hedgehog signaling is involved in cell proliferation and differentiation, invasion and metastasis, apoptosis, and angiogenesis, leading to tumorigenesis including medulloblastoma and basal cell carcinoma (BCC). *HYDIN* is a cilium-defect related gene that encodes *HYDIN* axonal receptor protein and is involved in Hedgehog signaling (53, 54). Therefore, it was possible to hypothesize that several genes associated with EOC were missing in patients with oviduct cilia loss. In the present study, it has been further demonstrated that cilia-associated genes were screened for relevant gene mutations, and this analysis showed that of the 20 candidate genes, four genes including HYDIN, PIK3CA, MUC6, PEDE4DIP had frequent mutations (>40%) in the EOC samples with tubal cilia loss. The majority of these genes were found to be involved in the regulation of tubulin and tubule assembly, or in the assembly of certain protein complexes of active cilia, among which the mutation rate of *HYDIN* was the highest. *HYDIN* is considered to be a key regulator of various malignant tumors in the human body and is involved in the invasion and metastasis of cancer cells (54). The WES analysis showed that a copy number loss of *HYDIN* existed in each of the 6 individuals with primary ciliary dyskinesia, and electron microscope scanning also confirmed that the cilia lacked in the *HYDIN* mutation samples (55). However, gene expression data alone is not able to determine the cell/subcellular localization of genes in tumor tissues. Accordingly, the positive expression of *HYDIN* detected by IHC analysis in ovarian cancer tissue and tubal cilia was further investigated. We found that the positive expression rate of *HYDIN* in the tube tissues and OC issues of EOC group was significantly lower than that of the Healthy control group. We hypothesized that oviduct ciliated cells and ovarian epithelial cells have similar regulatory genes, and the ciliated cells may have a role in the occurrence and susceptibility of EOC. Finally, RT-qPCR and western blotting analyses were used to detect the expression of *HYDIN* genes in normal ovarian tissue, ovarian cancer tissue, normal fallopian tube tissue, and EOC group fallopian tube tissue. These experiments also yielded consistent results, where essentially *HYDIN* expression was found to be markedly absent in ovarian cancer samples and fallopian tubes of EOC group.

In conclusion, in the present study, Cilia loss in cases of EOC were analyzed, in conjunction with an investigation to search for corresponding cilia-associated gene mutations. The preliminary research data obtained in this study have shown that the protein expression of *HYDIN* is decreased in ovarian and tubal cilia-deficient tissues. Moreover, the loss of *HYDIN* expression was closely associated with abnormal and shortened cilia. The decreased expression of *HYDIN* and other cilia-associated genes has an important role in inhibiting the formation of tubal cilia, which may promote the development of ovarian cancer. The specific mechanism can be further studied through *in vivo* and *in vitro* experiments. This study provides a better idea for the study of the pathogenic mechanism of EOC. Ciliated cells may be involved in the occurrence and development of EOC, and cilia-related gene HYDIN is expected to be a tumor marker for EOC.

## Data Availability

The datasets presented in this study can be found in online repositories. The names of the repository/repositories and accession number(s) can be found in the article/supplementary material.
